# Do metabolic factors increase the risk of thyroid cancer? a Mendelian randomization study

**DOI:** 10.3389/fendo.2023.1234000

**Published:** 2023-09-15

**Authors:** Weiwei Liang, FangFang Sun

**Affiliations:** ^1^ Department of Endocrinology, The Second Affiliated Hospital, Zhejiang University School of Medicine, Hangzhou, China; ^2^ Key Laboratory of Cancer Prevention and Intervention, China National Ministry of Education, The Second Affiliated Hospital, Cancer Institute, Zhejiang University School of Medicine, Hangzhou, China

**Keywords:** metabolic factors, HDL, thyroid cancer, Mendelian randomization, public health

## Abstract

**Background:**

Epidemiological studies emphasize the link between metabolic factors and thyroid cancer. Using Mendelian randomization (MR), we assessed the possible causal impact of metabolic factors on thyroid cancer for the first time.

**Methods:**

Summary statistics for metabolic factors and thyroid cancer were obtained from published Genome-wide association studies. The causal relationships were assessed using the inverse-variance weighted (IVW) method as the primary method through a two-sample Mendelian Randomization (MR) analysis. To account for the potential existence of horizontal pleiotropy, four additional methods were employed, including Mendelian Randomization–Egger (MR-Egger), weighted median method (WM), simple mode, and weighted mode method. Given the presence of interactions between metabolic factors, a multivariable MR analysis was subsequently conducted.

**Results:**

The results showed there was a genetic link between HDL level and protection effect of thyroid cancer using IVW (OR= 0.75, 95% confidence intervals [CIs] 0.60-0.93, p=0.01) and MR-Egger method (OR= 0.70, 95% confidence intervals [CIs] 0.50- 0.97, p=0.03). The results remained robust in multivariable MR analysis for the genetic link between HDL level and protection effect of thyroid cancer (OR= 0.74, 95% confidence intervals [CIs] 0.55-0.99, p=0.04).

**Conclusions:**

This study suggests a protection role for HDL on thyroid cancer. The study findings provide evidence for the public health suggestion for thyroid cancer prevention. HDL’s potential as a pharmacological target needs further validation.

## Introduction

Thyroid cancer is widely regarded as the most prevalent endocrine malignancy. In numerous countries, the frequency of thyroid cancer has experienced a notable rise in recent decades (1). The treatment options for thyroid cancer encompass surgical intervention to excise the thyroid gland, radioactive iodine therapy, and hormone replacement therapy. With early detection and appropriate treatment, patients have a good chance of long-term survival and a good quality of life. However, ongoing monitoring and follow-up care is important to detect any recurrence or new cancerous growths.

The exact cause of thyroid cancer is not known. Various studies have linked metabolic factors to thyroid cancer, but the majority of the findings remain controversial. There exists empirical evidence indicating that metabolic factors are associated with an elevated risk of developing various carcinogenic mechanisms, including those affecting the liver, colon, and mammary tissue, but the association between thyroid cancer and metabolic factors is inconsistent (2, 3). Specifically, the correlation between diabetes and thyroid cancer has yielded inconsistent results across studies (2, 3). Existing research posits that metabolic hormone imbalances, including insulin and leptin, may play a role in the pathogenesis of thyroid cancer (4, 5). Elevated insulin resistance and heightened insulin levels in the bloodstream have been correlated with an augmented susceptibility to thyroid cancer (4). Furthermore, obesity, which is concomitant with insulin resistance, has been demonstrated as a risk factor for the onset of thyroid cancer (6, 7), although this was not corroborated by a Mendelian randomization study (8). Additionally, reduced levels of vitamin D have been associated with an increased likelihood of thyroid cancer (9). Nevertheless, there exists evidence that vitamin D levels are not linked to the risk of thyroid cancer (10). A retrospective cohort study has reported a positive correlation between uric acid and thyroid nodules (11), while a cohort study from China has reported an association between nonalcoholic fatty liver disease and an increased risk of thyroid cancer (12). These studies are predominantly epidemiological and clinical in nature, and the causal relationship remains unclear. Therefore, it is imperative to evaluate the causality of these associations to inform updates to thyroid cancer prevention strategies.

The Mendelian randomization (MR) technique is a statistical methodology employed to investigate the causal associations between variables in observational research (13). It is based on the principle of Mendel’s laws of inheritance, which state that the distribution of genetic variations among offspring is random (14). Due to the random assignment of genotypes during the transmission from parents to offspring (14), it can be inferred that groups of individuals characterized by genetic variation related to a particular exposure at a population level are expected to have minimal association with the confounding factors commonly encountered in observational epidemiology studies. Furthermore, germline genetic variation remains unchanged after conception and is not influenced by the occurrence of any outcome or disease, thereby eliminating the possibility of reverse causation. The utilization of genetic variations as instrumental variables in MR enables the inference of the causal effect of a risk factor on a specific outcome of interest. Notably, MR offers an advantage over conventional observational studies by facilitating the establishment of a causal relationship between a risk factor and an outcome, despite the presence of confounding factors (15). Genetic variants that are correlated with the risk factor of interest are detected in MR studies and employed as surrogates for the exposure (16). These variants are then used to estimate the causal effect of the risk factor on the outcome, while controlling for the influence of other confounding variables. Because genetic variants are randomly assigned at conception, they are not subject to the biases and confounding factors that can impact the results of observational studies (17). Thus, Mendelian randomization can be conceptualized as akin to a randomized controlled trial conducted by nature. The MR method has become a popular tool in epidemiology and public health research, particularly for investigating the causal relationships between lifestyle factors and health outcomes. The results of MR studies have provided valuable insights into the causal relationships between risk factors and health outcomes and have helped to inform public health policies and interventions aimed at improving population health (18).

In this article, we applied Mendelian randomization methodology to explore the causal association between metabolic factors and thyroid cancer.

## Methods

**Figure 1:**
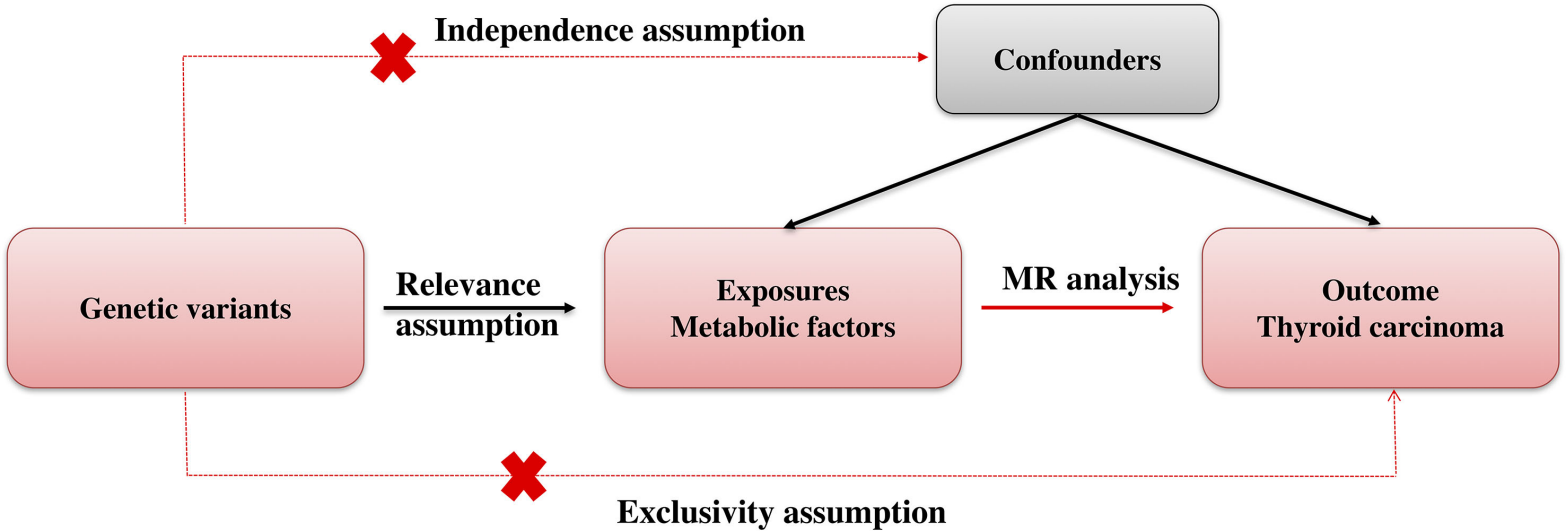
Experimental workflow. MR, Mendelian randomization.

Mendelian randomization (MR) employs genetic variation as a means to investigate causal inquiries pertaining to the potential impact of modifiable exposures on health, developmental, or social outcomes. Methods for MR are usually based on instrumental variables (IVs). Genetic variants serve as a potential exogenous source of variation in the exposure, thereby functioning as an IV. **Figure 1** showed our study workflow.

We tried to cover metabolic factors as much as we can to provide evidence for the public health suggestion for thyroid cancer prevention. Metabolic factors reported to be associated with thyroid cancer but that remained controversial were included in the study (2–12). Metabolic factors reported in other solid tumors but lack of evidence in thyroid cancer were also included in the study (19–21). Finally, our study included 17 metabolic factors according to present epidemic study reporting the relevance to thyroid cancer. Firstly, we estimated the associations of metabolic factors and thyroid cancer using univariable MR analysis. Considering the metabolic factors may have interaction, multivariable MR analysis were conducted to increase the analysis power. The study was based on publicly available, summary-level data of genome- wide association studies (GWAS), the FinnGen study (22), the UK Biobank study (23), and other large consortia. Informed consent was obtained from participants in included studies, which were approved by an appropriate ethical review board.

### Exposures chosen

Significant SNPs for 17 metabolic factors were extracted from corresponding GWAS studies (**Table 1**). The SNP used as the exposure instrumental variables (IVs) were selected with a p-value less than 5E-8. Then we performed linkage disequilibria based clumping to return only independent significant associations. SNPs without linkage disequilibrium r2 < 0.001 and a clump distance >10,000kb window were obtained.

**Table 1:** Metabolic factors included in the Mendelian randomization study.

Exposure	Participants Included in Analysis	Dataset
Body mass index	339,224	ieu-a-2
Height	6,974	ieu-a-1032
Waist-to-hip ratio	224,459	ieu-a-72
Body fat	100,716	ieu-a-999
LDL cholesterol	440,546	ieu-b-110
HDL cholesterol	403,943	ieu-b-109
triglycerides	441,016	ieu-b-111
Total cholesterol	187,365	ieu-a-301
apolipoprotein A-I	393,193	ieu-b-107
Adiponectin	39,883	ieu-a-1
Nonalcoholic fatty liver disease	218792	finn-b-NAFLD
Type 2 diabetes	655,666	ebi-a-GCST006867
Hemoglobin A1c	42,790	bbj-a-26
Serum 25-Hydroxyvitamin D levels	496,946	ebi-a-GCST90000618
Uric acid	109,029	bbj-a-57
hypertension	463,010	ukb-b-12493
Systolic blood pressure	757,601	ieu-b-38
diastolic blood pressure	757,601	ieu-b-39

### Outcomes chosen

Based on reported GWAS data, we obtained summary statistics on SNP associations with thyroid cancer. GWAS data from the largest publicly available thyroid cancer case–control study involving 218792 Europeans (989 cases, 217,803 controls) was obtained from FinnGen. 16,380,466 SNPs in finn-b-C3_THYROID_GLAND was downloaded for further analysis.

### Statistical analysis

IVs and outcome data were firstly harmonized to be relative to the same allele. MR analysis was then conducted. Various methods were employed to assess the resilience of the outcomes and identify pleiotropy, such as the inverse-variance weighted (IVW), Mendelian Randomization–Egger (MR-Egger), weighted median method (WM), simple mode, and weighted mode method, in order to compute the causal effect. Analyzing causal relationships was primarily conducted using IVW methods. Results were mostly derived from IVW (random effects) and sensitivity analysis. The meta-analysis approach employed by IVW amalgamates the Wald ratios of individual SNPs to yield precise estimates. A significance level of P < 0.05 was deemed indicative of a potential association. The MR-Egger method is a proficient strategy for identifying deviations from the assumptions underlying instrumental variables (24). Weighted median method can provide sensitivity analyses with multiple genetic variants. If the weight of valid instruments exceeds 50%, consistent causal estimates may be obtained (25). Although less powerful than IVW, simple mode offers robustness against pleiotropy (26). As a supplementary analysis method, weighted mode is sensitive to challenging bandwidth selections for mode estimation (27). The MR-Egger regression intercept term tests were utilized to identify horizontal pleiotropy. Heterogeneity in IVW and MR-Egger regression analyses was quantified using Cochran’s test.

For significant associations identified in the analyses, the multivariable MR was further used as a sensitivity analysis to explore whether this causal effect was robust to the adjustment.

All statistical analyses were conducted in R (version 4.2.2) using the TwoSampleMR (28), MRInstruments packages. Plots were generated using ggplot2 R package. Our code is publicly available on GitHub: https://github.com/heleliangww/MR-for-thyroid-cancer-.

## Result

**Figure 2:**
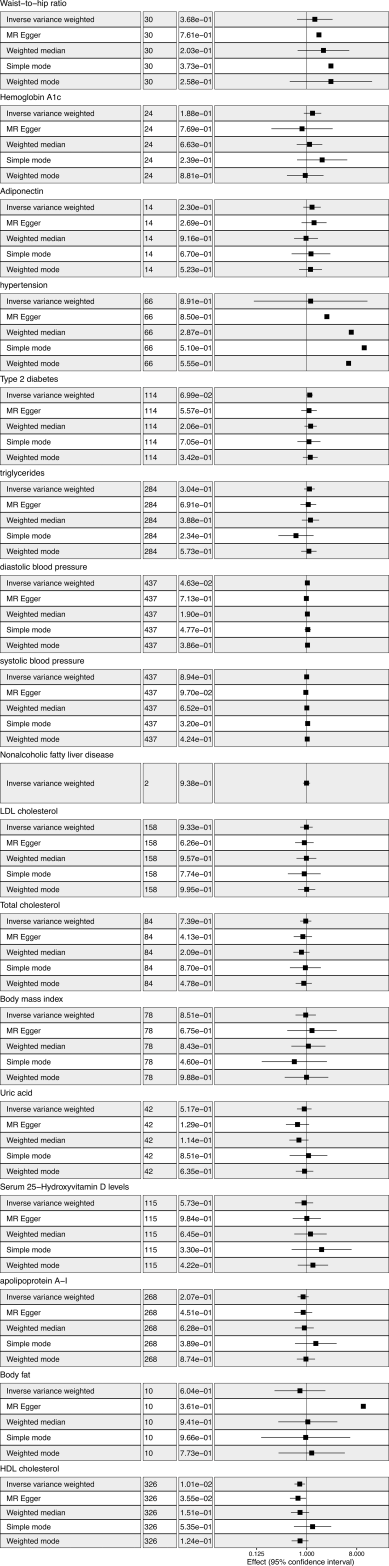
Metabolic factors and thyroid cancer in Mendelian randomization (MR) analyses. The first column from left showed the corresponding methods. The second column from left showed the number of SNPs involved in the analyses. The third column from left showed the corresponding *p* value. The forth column from left showed odds ratio and 95% confidence interval.

**Figure 3:**
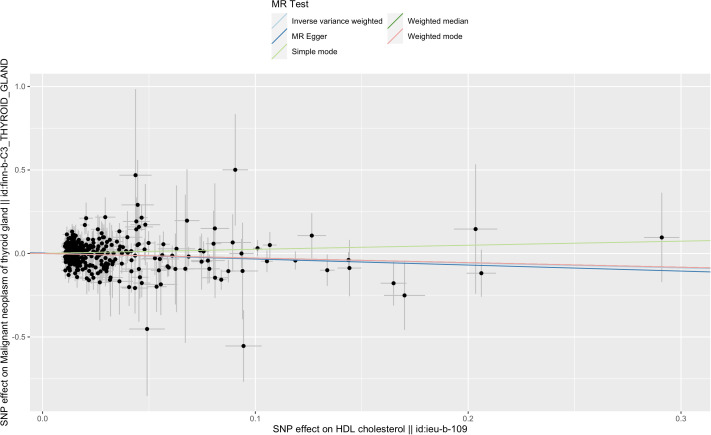
The scatter plot of five Mendelian randomization methods on HDL and thyroid cancer.

**Figure 4:**
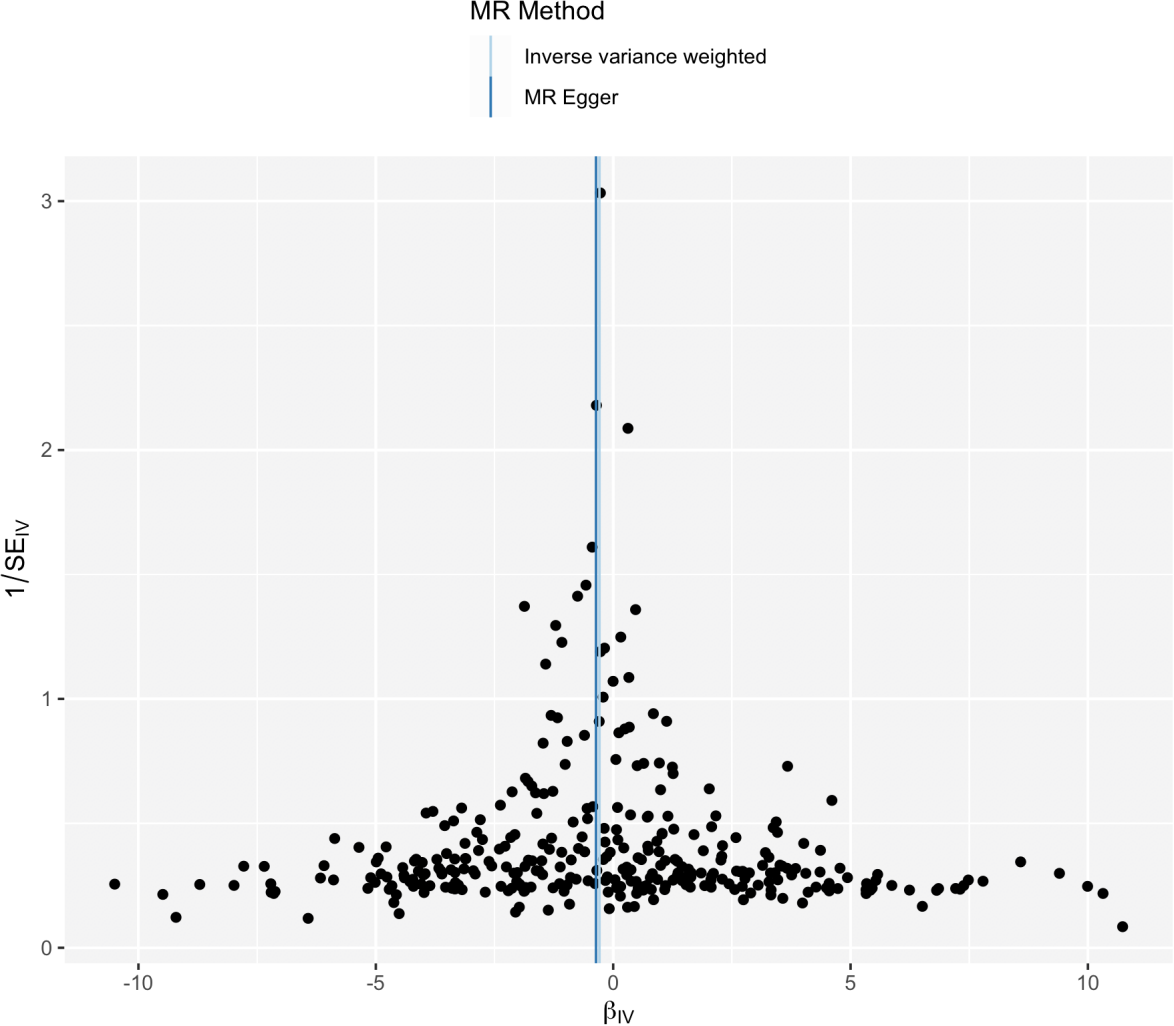
Funnel plot for HDL and thyroid cancer.

IVW analysis showed there was a genetic link between HDL level and protection effect of thyroid cancer (**Figure 2**). Results revealed an increase in HDL level was strongly associated with a decrease in the risk of thyroid cancer (OR= 0.75, 95% confidence intervals [CIs] 0.60-0.93, p=0.01). The scatter plots in **Figure 3** illustrated the SNP- thyroid cancer associations against the SNP-HDL associations. There was a consistent association in sensitivity analyses using MR-Egger method (OR= 0.70, 95% confidence intervals [CIs] 0.50- 0.97, p=0.03). Based on MR-Egger regression intercept analysis, no significant horizontal pleiotropy was detected (intercept= 0.002, SE= 0.005, p= 0.58). Using Cochran’s Q test, no heterogeneity was observed among SNPs in IVW analysis and MR-Egger analysis, suggesting no strong unbalanced horizontal pleiotropy (Q_pval = 0.09 in IVW method, Q_pval= 0.09 in MR Egger method). There was a balanced pleiotropy in SNP effects around the effect estimate, as evidenced by the funnel plot (**Figure 4**).

IVW analysis showed there was a genetic link between diastolic blood pressure and increased risk of thyroid cancer (**Figure 2**). Results revealed an increase in diastolic blood pressure level may associated with an increase in the risk of thyroid cancer (OR=1.03, 95% confidence intervals [CIs] 1.00-1.06, p=0.046). While, the result was not consistent in MR-Egger method analysis (OR= 0.99, 95% confidence intervals [CIs] 0.92- 1.06, p=0.71).

**Figure 5:**
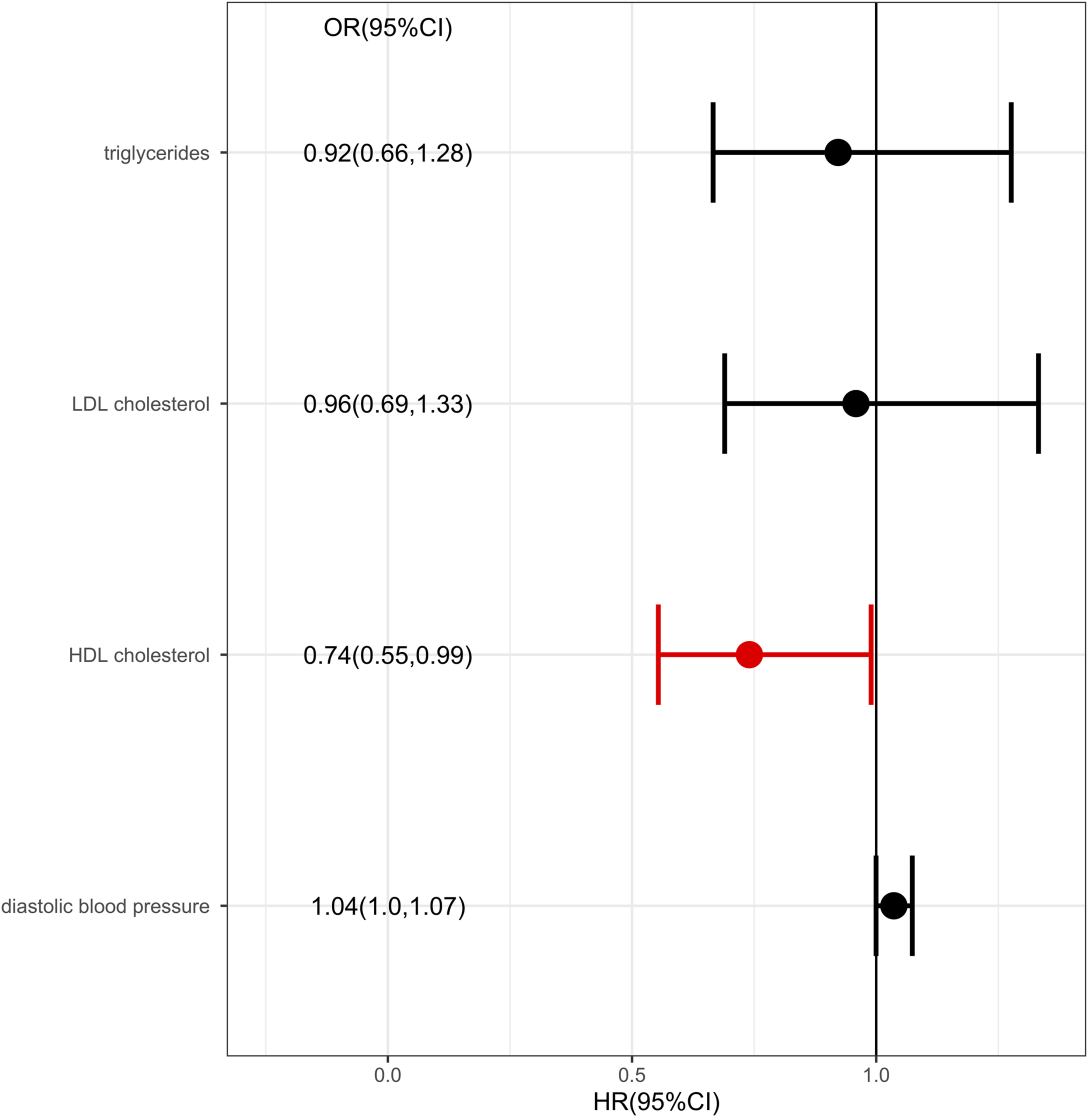
Multivariable MR result for metabolic factors and thyroid cancer.

Considering there were interactions between different lipid components, multivariable MR was conducted. Diastolic blood pressure was also included in the multivariable MR analysis for its positive IVW analysis (**Figure 5**). As with the univariate MR analysis, the results remained robust in multivariable MR analysis for the genetic link between HDL level and protection effect of thyroid cancer (OR= 0.74, 95% confidence intervals [CIs] 0.55-0.99, p=0.04).

## Discussion

This study used GWAS summary statistics to perform MR analysis to investigate the causal association between thyroid cancer and metabolic factors. We believe this is the first MR study to identify a large number of modifiable causal risk factors for thyroid cancer. We found serum HDL-cholesterol level was associated with a reduced risk of thyroid cancer. We did not find a causal relationship between obesity, diabetes, blood pressure, NAFLD, uric acid, and serum 25-hydroxyvitamin D levels and thyroid cancer.

HDL, also known as high-density lipoprotein, is commonly acknowledged as “good” cholesterol due to its ability to eliminate excess cholesterol from the bloodstream and transport it to the liver for processing and excretion from the body. Numerous published observational studies have established a consistent correlation between HDL and thyroid cancer. For instance, a Korean epidemiological study discovered that obese women with low HDL cholesterol levels were at a heightened risk of developing thyroid cancer (29, 30). Similarly, the Swedish Apolipoprotein-Related Mortality Risk (AMORIS) Cohort study demonstrated that thyroid cancer risk was associated with blood levels of total cholesterol (TC) and HDL-C (31). HDL-C level was found to be a statistically significant independent predictor of thyroid cancer in a model developed by Zhang et al. (32). Some retrospective observational studies have reported an association between total cholesterol (31) and apolipoprotein A1 (33) with thyroid cancer, which is somewhat inconsistent with the results of our study. In the observational study, HDL may be a confounding factor for other lipid profiles.

Few studies have investigated the mechanism of HDL in thyroid cancer *in vivo* and *in vitro*. HDL has been reported to play a role in the invasion, metastasis, and development of other solid tumors. When HDL levels are within a certain range, tumor development can be inhibited *in vivo (* 34). *In vitro* studies have shown that HDL inhibits tumor cell growth or promotes apoptosis by inhibiting components of tumor microenvironments (34). The HDL reduce oxidative stress and proinflammatory molecules in cancer cells (35). Additionally, HDL can inhibit angiogenesis and reverse tumor immune escape (35). In pancreatic ductal adenocarcinoma, research showed cancer cell growth is reduced by HDL-mediated cholesterol removal (36). Relevant functional studies are lacking, further research is needed to fully understand the relationship between HDL and thyroid cancer.

IVW analysis showed a genetic link between diastolic blood pressure and thyroid cancer, which was inconsistent in MR-Egger method analysis. The result might be biased by pleiotropy or other confounding factors.

Unlike observational studies, our results do not confirm a causal role for other metabolic factors in thyroid cancer. Confounding factors such as HDL levels may lead to false associations in clinical observations. By using genetic variants, we can limit those confounding factors in by using Mendelian randomization.

In this study, we address metabolic factors and related traits and the effect on thyroid cancer for the first time using Mendelian randomization. We acknowledge, however, that there are some limitations to our study. Our MR analysis power was limited by the fact that we had only 989 thyroid cancer cases. Our analysis was not stratified by gender. There is a need for further GWAS studies with a larger number of cases and detailed information on disease characteristics.

In conclusion, our study found serum HDL-cholesterol level was associated with a reduced risk of thyroid cancer. Our study provided genetic evidence that HDL might protect thyroid cancer patients. The study findings provide evidence for the public health suggestion for thyroid cancer prevention. Further validation of our findings in other cohorts and ethnicities will require independent GWAS and large prospective studies. HDL’s potential as a pharmacological target needs further validation.

## Data availability statement

Publicly available datasets were analyzed in this study. This data can be found here: The study was based on publicly available, summary-level data of genome- wide association studies (GWAS), the FinnGen study, the UK Biobank study.

## Ethics statement

Ethical approval was not required for the study involving humans in accordance with the local legislation and institutional requirements. Written informed consent to participate in this study was not required from the participants or the participants’ legal guardians/next of kin in accordance with the national legislation and the institutional requirements.

## Author contributions

WL made significant contributions to the literature search and study design, as well as the analysis and interpretation of the data, ultimately resulting in the composition of the manuscript. FS, on the other hand, played a crucial role in formatting the figures and tables, as well as revising the manuscript. Additionally, FS provided valuable insights and constructive discussions during the analysis process. All authors contributed to the article and approved the submitted version.
